# Percutaneous mastoid electrical stimulator improves Poststroke depression and cognitive function in patients with Ischaemic stroke: a prospective, randomized, double-blind, and sham-controlled study

**DOI:** 10.1186/s12883-020-01795-0

**Published:** 2020-05-29

**Authors:** Taoli Lu, Lanying He, Bei Zhang, Jian Wang, Lili Zhang, Wei Wei Dong, Hao Yang

**Affiliations:** 1 Department of Neurology, The Second People’s Hospital of Chengdu, Chengdu, 610021 PR China; 2 Department of Neurology, First Affiliated Hospital, Chongqing Medical University, Chongqing, 400030 PR China; 3 College of Electrical Engineering, Institute of Electrical Technology, Chongqing University, Chongqing, 400030 PR China

**Keywords:** Acute ischaemic stroke, Percutaneous mastoid electrical stimulator, Poststroke depression, Cognition

## Abstract

**Background:**

Poststroke depression can lead to functional dependence, cognitive impairment and reduced quality of life. The aim of this study was to evaluate the effects of a percutaneous mastoid electrical stimulator (PMES) plus antidepressants on poststroke depression and cognitive function.

**Methods:**

This study was a prospective, randomized, double-blind, and sham-controlled study. A total of 258 clinically depressed ischaemic stroke patients within 14 days of index stroke were randomly assigned to the PMES plus antidepressant (PMES group, *N* = 125) and sham plus antidepressant (sham group, *N* = 133) groups. All patients underwent the Montreal Cognitive Assessment (MoCA) and Hamilton Rating Scale for Depression (HRSD) test at 2 weeks (baseline), and 6 months(M6) after ischaemic stroke. Primary outcomes were the percentage of patients showing a treatment response (≥50% reduction in HRSD score) and depression remission (HRSD score ≤ 9) at 6 months. The secondary outcome was the percentage of patients with a MoCA score < 26.

**Results:**

The percentages of patients showing a treatment response and depression remission were significantly higher in the PMES group than in the sham group (57.60% vs 41.35%, *P* = 0.009; 44.00% vs 29.32%, *P* = 0.014 respectively). The mean value of the HRSD score change [M (month)6-baseline] was significantly higher in the PMES group than in the sham group at 6 months (− 11.93 ± 5.32 vs − 10.48 ± 6.10, *P* = 0.036, respectively). The percentage of patients with MoCA scores < 26 was lower in the PEMS group than in the sham group (12.0% vs 24.06%, *P* = 0.012,respectively), and the mean value of the MoCA score change (M6-baseline) was higher in the PMES group than in the sham group (3.50 ± 2.55 vs 2.72 ± 2.52, *P* = 0.005, respectively).

**Conclusion:**

These findings demonstrate that PMES adjunctive to antidepressant therapy is effective in reducing depression, achieving remission in the short term, and improving cognition.

**Trial registration:**

This trial was retrospectively registered (registration number: ChiCTR1800016463) on 03 June 2018.

## Background

Stroke is a leading cause of long-term disability. Despite impressive progress in early diagnosis and medical treatment, which has resulted in a decrease in the incidence and mortality rates of stroke, approximately 25–74% of stroke patients still suffer major disability and psychological illness, including depression, cognitive impairment, and social isolation [1, 2]. Poststroke depression PSD) is associated with poor outcomes after stroke, including cognitive disorders, and poor rehabilitation outcomes [3, 4]. PSD has a prevalence of approximately 30% in stroke survivors based on previous studies [5].

The treatment of PSD includes medication and psychotherapy [6–11]. Selective serotonin reuptake inhibitors (SSRIs) are the most commonly used drugs in the treatment of PSD [6–8]. However, some patients are reported to experience insufficient efficacy and adverse events. Psychotherapy has a poor effect on PSD. Hence, it is very important to find a non-pharmacologic treatment for PSD [10, 11].

In 1998, neuroprotection with fastigial nucleus stimulation (FNS) was first confirmed by Reis [12], and the results showed that 1 h of FNS treatment in anaesthetized rats prior to middle cerebral artery occlusion (MCAO) reduced the volume of the focal infarction by 50%. In recent decades, many studies have shown that FNS has a variety of neuroprotective mechanisms [13]. FNS can inhibit the electrical activity around the focus, reduce the excitotoxic injury of neurons, inhibit the inflammatory response, and inhibit apoptosis [13].

Non-invasive percutaneous mastoid electrical stimulator (PMES) is called cerebrovascular function therapy (CVFT) device in China, and uses a biological bionic current to therapeutically stimulate the fastigial nucleus (FN). It is demonstrated by animal experiments that FN stimulation can be achieved extracranially [14]. During electrical stimulation, excited nerve fibres pass through the FN, resulting in increased blood pressure, reflexive vasodilatation and increased cerebral blood flow (CBF), which, taken together, is called the fastigial pressor response (FPR) [15]. By inhibiting the baroreceptor reflex, the FPR is enhanced, and adrenaline, noradrenaline and arginine vasopressin are released [16, 17]. The increase in CBF is global (including the spinal cord), and the largest increases are in the frontal lobe and parasagittal area of the cortex [18, 19]. Fastigial nucleus stimulation can induce neuroprotection against cerebral ischaemia, and electrical stimulation of the cerebellar dentate nucleus or white matter does not have a neuroprotective effect. In addition, FNS treatment after selective injury of FN neurons failed to induce neuroprotection, suggesting that the protection of FNS to cerebral ischaemia was generated in the intrinsic FN neurons [20].

FNS has been reported to improve depression and cognitive function after stroke in animal experiments [21–23]. Some observational studies have shown that PMES treatment can improve clinical prognoses and has a good safety profile [24–27]. However, due to the small number of patients in these studies, there remains a lack of evidence regarding the clinical efficacy of FNS in PSD. The purpose of this study was to explore the effect of PMES combined with antidepressants on PSD and cognitive function.

## Methods

### Study population

This study was a prospective, randomized, double-blind, and sham-controlled study. This project was registered in the Chinese Clinical Trial Register (ChiCTR) (the registration for trial number ChiCTR1800016463 was retrospectively completed on June 32,018) and was performed according to the CONSORT 2010 extension to randomized pilot and feasibility trials [28]. The patients were admitted to the Second People’s Hospital of Chengdu due to ischaemic stroke within 14 days of symptom onset between January 2015 and December 2018. Ischaemic stroke was confirmed by brain computed tomography or magnetic resonance imaging.

### Depression screening

Potential participants screened positive for depressive symptoms, and had a diagnosis of clinical depression that was verified by a diagnostic interview using DSM-V criteria. Depression screening was carried out by the 30-item Geriatric Depression Scale (GDS), which consists of 30 questions, that are individually scored as 1 point, resulting in a range of 0–30 points that were classified as follows: 0–10, no depression; 11–20, mild depression; 21–30, moderate depression. The diagnosis of depression was validated by the Hamilton Rating Scale for Depression (HRSD) in those who scored ≥11 on the GDS and consented to the full study. Stroke severity was assessed based on the National Institutes of Health Stroke Scale (NIHSS). The study was approved by the ethics committees of the Second People’s Hospital of Chengdu. Informed consent was signed by all the participants.

### Inclusion and exclusion criteria

Patients were included if they fulfilled all the following criteria: (1) admission for first-ever ischaemic stroke within 14 days, (2) no neurological or psychiatric disease before stroke, (3) no aphasia,(4) no drug abuse, (5) no severe hearing deficit, (6) right-handed, (7) no serious dysarthria and (8) able to cooperate,(9) no active malignancies, and (10) capable of appropriate communication.

### Study design and grouping

The patients were divided into two groups: the sham and PMES groups. The patients in the PMES group received PMES treatment as an add-on to antidepressant treatment and the patients in sham group received sham stimulation and antidepressant treatment.

### Treatment methods

**Fig. 1:**
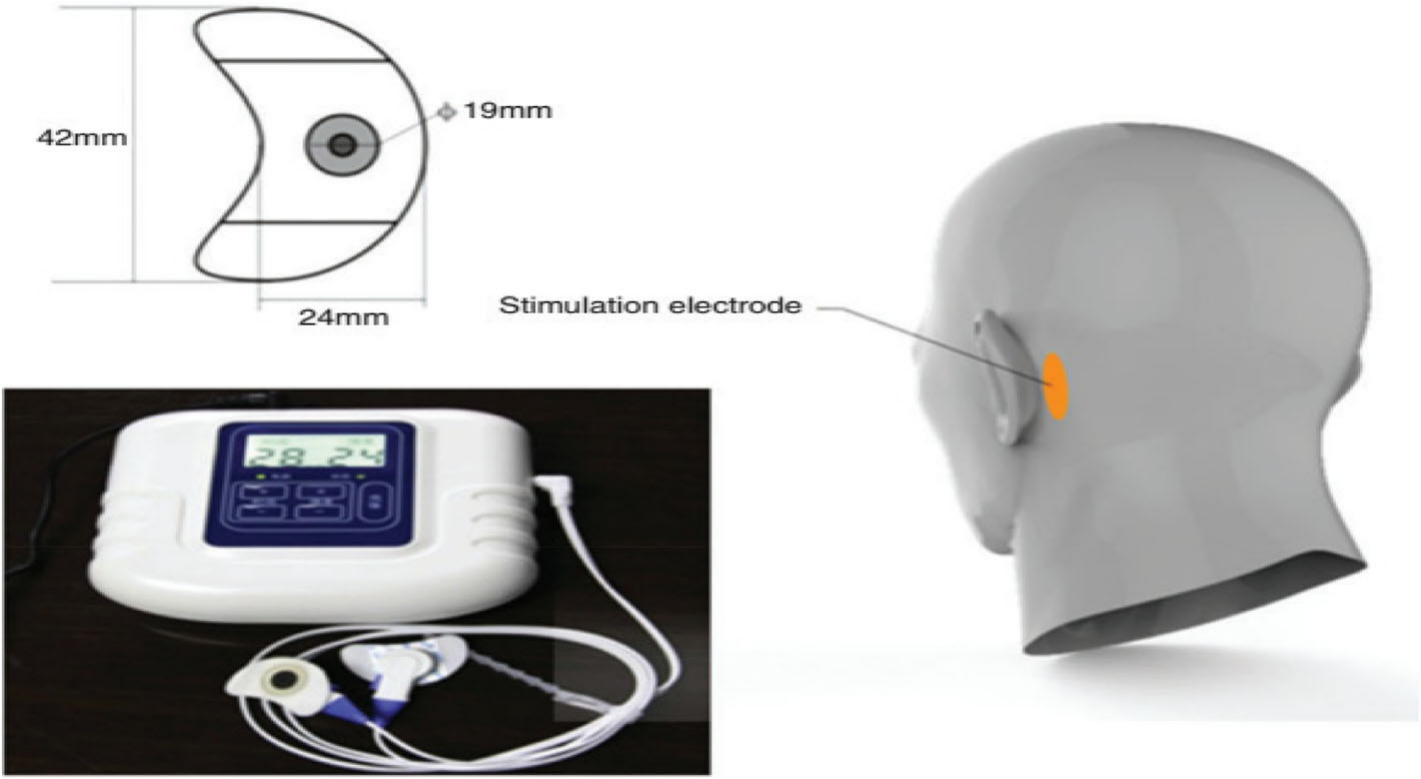
The percutaneous mastoid electrical stimulator (PMES) device and stimulation electrode placed on mastoid area behind the ear (Each of the images included in figure 1 are your own)

The PMES and sham treatment methods were the same as those used in our previous work [29, 30]. The bilateral mastoid skin was cleaned, and then the stimulation electrodes were placed. The sizes of electrode and conductive gel were 42 × 24 mm and 19 mm, respectively (Fig. 1, [29]). The stimulus parameters were as follows: pulse width of 90 mS for both PMES and sham, frequencies of 1.8 kHz for PMES and 10 Hz for sham, peak currents of 10 mA for PMES and 0.18 mA for sham [29]. On the basis of previous studies, we found that 10 mA was safe, and some patients experienced mild tingling but no skin redness or burns [29]. To reduce the surface sensations caused by current stimulation, the low-frequency signal (13–45 Hz) was modulated to the intermediate frequency signal of 1.8 kHz, and the voltage range was 1.0–1.2 v [29]. The intermediate-frequency signal was the exponential decay signal with a base of “a” (0 < a < 1). The signal was a nonpolar exponential wave, which was composed of a positive pulse, a negative pulse wave and an equivalent charge. The negative pulse depolarizes the nerve fibre, and the positive pulse balances the charge, which can eliminate the accumulation of electrostatic charge and reduce adverse electrochemical reactions. To reduce the energy of a single pulse, we reduce the base value “a”. The surface sensations from the PMES stimulus were close to those of the sham stimulus, which was a periodic point-contact sense of touch. The PMES group and sham group were treated for 45 min/day for 6 months.

In this study, selective serotonin reuptake inhibitor (SSRI) were recommended as the first choice for depressive patients, and sertraline was recommended as the initial antidepressant because of its tolerance to medical treatment and relatively low incidence of cardiovascular side effects. The patients were prescribed sertraline 50 mg/day, and the dose was adjusted starting from day 7 to 100 mg/day (maximum dose: 400 mg/day). If patients could not tolerate the side effects of sertraline, another antidepressant was prescribed (escitalopram or paroxetine).

### Randomization and double blinding

The patients who met the criteria were assigned to treatment groups according to a predefined randomization plan by using a block size of 4, a ratio of 1:1, and stratified by study team. A computer-generated block randomization list was prepared by the Clinical Research Unit of The Second People’s Hospital of Chengdu. The randomization was conducted by a statistical analyser who was not involved in other parts of the study. The patients, investigators and all study personnel were blinded to the treatment allocation. The PMES and sham stimulators had the same external appearances, user manuals and electrodes. They could not be distinguished by their external appearance. We took the following measures to guarantee double-blinding: enrolled patients were not acquainted with each other, there was no physical contact or communication (such as sensory perception) between patients during visits, and all of the patients would be told when enrolled that it was not possible to accurately judge whether they were receiving true or sham stimulation based only on the surface sensations.

### Data collection

Baseline characteristics included demographics, stroke characteristics, NIHSS score, and risk factors. All patients underwent depressive state and cognitive assessment at 2 weeks (baseline) and 6 months after ischaemic stroke.

Depressive states were assessed using HRSD scores. Treatment responses were defined as ≥50% reduction in the HRSD score. Remission was variably defined as an HRSD score of ≤9 (no longer meeting the depression criterion), ≤7 (absence of any depressive symptoms), or ≤ 3 (equivalent to healthy controls). We used HRSD scores of ≤9 and a ≥ 50% reduction in HRSD scores for comparison with baseline. Cognitive status was assessed using the Montreal Cognitive Assessment (MoCA), with scores that can range from 0 to 30 points, with lower scores reflecting greater cognitive impairment, and a cut-off of < 26 was considered indicative of cognitive impairment.

All patients were followed up for 6 months. After discharge, the patients completed treatment at home or in a nursing home. The patients or caregivers in both groups were trained in using the PMES and sham stimulators. All patients were followed up once a month by face-to-face interviews or telephone interviews.

Changes in HRSD and MoCA scores were detected at 6 months after treatment. Primary outcomes were treatment response (≥50% reduction in HRSD score) and depression remission (HRSD score ≤ 9) at 6 months after ischaemic stroke. The secondary outcome was the percentage of patients with a 6-month MoCA score < 26.

### Statistical analysis

The treatment response rates in the PMES group and sham group were approximately 55 and 35%, respectively. To examine the significant difference between these two groups, the bilateral significance level was established at 5%, and the power of the test was 80%. Considering a 20% loss to follow-up, the sample size of each group was estimated at approximately 120 cases.

Demographic characteristics and vascular risk factors were compared between the sham and PMES groups. Continuous data were expressed as the mean values (±standard deviation); using the Mann–Whiney U test. Categorical data were described using frequency and percentage, and compared using the Pearson χ2 test, or Fisher’s exact 2-sided test. The data were analysed using SPSS software (SPSS 22.0). *P* values< 0.05 were considered statistically significant.

## Result

### Characteristics of the study subjects

**Fig. 2:**
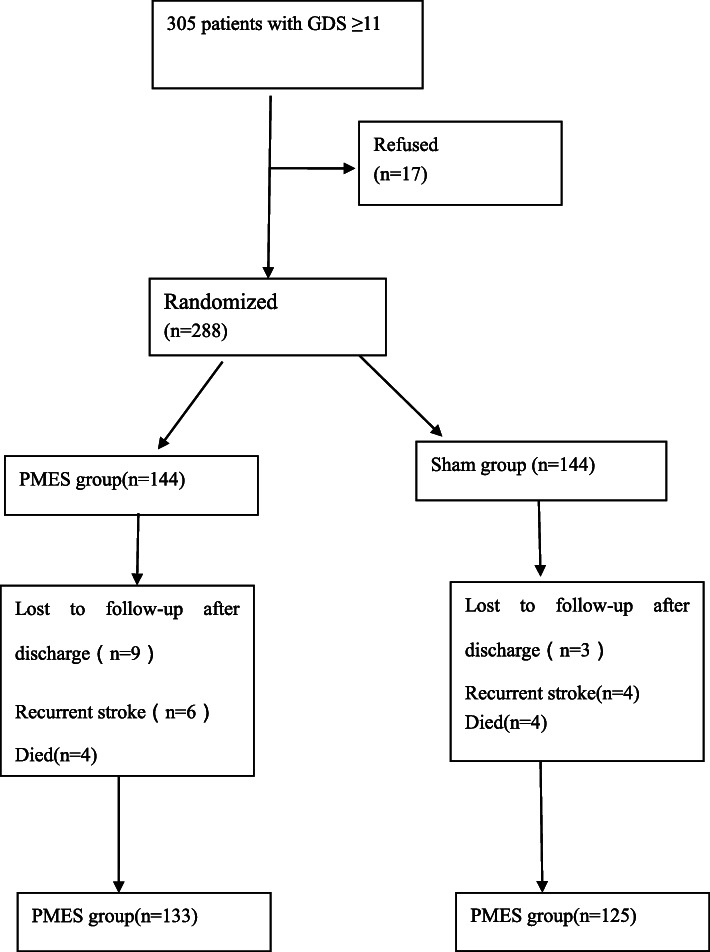
Patient’s flowchart

Approximately 1000 patients with ischaemic stroke were tracked for potential screening eligibility. Some patients were not eligible (aphasia, severe hearing deficit, psychiatric disease before stroke, drug abuse). A total of 810 patients agreed to be screened. A total of 305 patients were eligible (GDS ≥11), 17 patients refused, and 288 patients were enrolled (See Fig. 2 for details on exclusions).

A total of 288 patients were enrolled in this study (sham group, *N* = 144; PMES group, *N* = 144). Twelve patients were lost to follow-up after discharge from the hospital (sham group, *N* = 3; PMES group, *N* = 9), 10 patients experienced recurrent stroke (sham group, *N* = 4; PMES group, *N* = 6), and 8 patients died during the 6-month follow-up period (sham group, *N* = 4; PMES group, *N* = 4). A total of 258 patients were finally analysed (sham group, *N* = 133; PMES group, *N* = 125) (Fig. 1), comprising 52.33% (135) men and 47.67% (123) women, the mean age was 65.58 ± 8.59 years (range:42–87 years). In the study population, 148 patients had a history of hypertension, 97 patients had a history of diabetes, 139 patients had a history of hyperlipidemia, and 91 patients smoked. The PMES and sham groups received treatment daily for 45 min, and the treatment lasted 6 months. There were no adverse reactions reported either in the PMES group or in the sham group during the treatment period.

Baseline characteristics of the patients in the sham group and the PMES group were compared (Table 1). Sertraline, escitalopram and paroxetine were the most commonly prescribed SSRI drugs. No patients stopped taking antidepressants during the follow-up period. There were also no significant group differences in the baseline HRSD and MoCA scores (P>0.05).

**Table 1:** Comparison of baseline characteristics at admission between patients with Sham and PMES groups

	Sham group (133)	PMES group (125)	OR(95%CI)	P*
Age, y (Mean SD)	66.11 ± 8.37	65.0 ± 8.82		0.622
NIHSS, (Mean SD)	6.99 ± 2.47	7.02 ± 2.21		0.978
Females, n(%)	68 (51.13)	55 (44.00)	0.751 (0.46–1.23)	0.465
Men, n(%)	65 (48.87)	70 (56.00)	0.751 (0.46–1.23)	0.252
BMI ≥ 24 kg/m, n(%)	32 (24.06)	42 (33.60)	1.60 (0.93–2.75)	0.090
Hypertension, n(%)	72 (54.14)	76 (60.8)	1.31 (0.80–2.16)	0.279
Current Smoking, n(%)	48 (36.09)	43 (34.40)	0.93 (0.56–1.55)	0.776
Current Drinking, n(%)	43 (32.33)	44 (33.08)	1.14 (0.68–1.91)	0.626
Diabetes, n(%)	54 (40.60)	43 (34.40)	0.77 (0.46–1.27)	0.304
Hyperlipidemia, n(%)	65 (48.87)	74 (59.20)	1.52 (0.93–2.45)	0.096
Atrial fibrillation, n(%)	50 (37.59)	40 (30.08)	0.78 (0.47–1.31)	0.346
Family history of stroke, n(%)	29 (21.80)	33 (26.40)	1.29 (0.73–2.28)	0.388
MoCA Score, (mean SD)	24.90 ± 3.16	24.90 ± 2.82		0.936
HRSD Score, (mean SD)	22.02 ± 4.54	21.51 ± 4.32		0.280
Medications use				
Antiplatelet, n(%)	43 (32.33)	48 (38.40)	1.31 (0.78–2.17)	0.308
Antihypertensive, n(%)	56 (42.11)	56 (44.80)	1.12 (0.68–1.83)	0.663
lipid-lowering medications, n(%)	64 (48.12)	71 (56.80)	1.42 (0.87–2.32)	0.163
Sertraline, n(%)	83 (62.41)	81 (60.90)	1.11 (0.67–1.84)	0.690
Escitalopram, n(%)	14 (10.53)	19 (14.29)	1.52 (0.73–3.19)	0.261
Paroxetine, n(%)	36 (27.07)	25 (18.80)	0.67 (0.38–1.21)	0.182
Infarct location				
Basal ganglia, n(%)	62 (46.62)	61 (45.86)	1.09 (0.67–1.78)	0.726
Brain stem, n(%)	18 (13.53)	20 (15.04)	1.22 (0.611–2.43)	0.576
Cerebellum, n(%)	10 (7.52)	4 (3.01)	0.41 (0.12–1.33)	0.126
Frontal lobe, n(%)	19 (14.29)	15 (11.28)	0.82 (0.40–1.69)	0.588
Parietal lobe, n(%)	10 (7.52)	9 (6.77)	0.95 (0.37–2.43)	0.922
Temporal lobe, n(%)	5 (3.76)	10 (8.00)	2.23 (0.74–6.71)	0.146
Occipital lobe, n(%)	9 (6.77)	6 (4.80)	0.70 (0.24–2.01)	0.500

*BMI* Body Mass Index, *SD* Standard deviation

*Comparison between sham and PMES groups. Demographic characteristics were compared between the 2 subgroups in univariate analysis, using Pearson χ2 test, Fisher exact 2-sided test, mean values(±standard deviation) were calculated for continuous variables. Mann-Whitney U test was used to test differences between two group

### Primary outcomes

There was no difference in the HRSD scores at baseline between the sham and PEMS groups (22.02 ± 4.54 vs 21.51 ± 4.32, *P* = 0.280, respectively) (Table 1). At the end of the 6-month intervention period, the HRSD score improved both in the sham and PMES groups (Table 2). The HRSD score was lower in PEMS than in the sham group (9.58 ± 3.45 vs 11.54 ± 4.21, *P* **<** 0.001, respectively), and the mean value of the HRSD score change (M6-baseline) was significantly greater in the PMES group than in the sham group at 6 months (− 11.93 ± 5.32 vs − 10.48 ± 6.10, *P* = 0.036, respectively) (Table 4).

**Table 2:** The mean value of the MoCA Score and HRSD at 6 months in Sham and PMES groups

	Sham group(133)	PMES group(125)	P*
MoCA Score, (Mean SD)	27.26 ± 2.20	28.26 ± 1.95	**< 0.001**
HRSD Score, (Mean SD)	11.54 ± 4.21	9.58 ± 3.45	**< 0.001**

Bold indicates *P*-values less than 0.05

*Continuous variables are expressed as mean ± standard deviation. Mann-Whitney U test was used to test differences between two groups. Categorical data were described using frequency and percentage, using Pearson χ2 test, Fisher exact 2-sided test

During the 6-month follow-up period, 126 patients showed a treatment response, and 94 patients showed depression remission (Table 3). The treatment response in the sham group was 41.35% (55/133) at 6 months, which was significantly lower than in the PMES group (57.60%, 72/125) (*P* = 0.009). Depression remission in the sham group was 29.32% (39/133) at 6 months, which was significantly lower than in the PMES group (44.00%, 55/125) (*P* = 0.014).

**Table 3:** The percentage of treatment response and depression remission in Sham and PMES groups

	Sham group(133)	PMES group(125)	OR(95%CI)	P*
Treatment response, n(%)	55 (41.35%)	72 (57.60%)	1.93 (1.18–3.16)	**0.009**
Depression remission, n(%)	39 (29.32%)	55 (44.00%)	1.89 (1.13–3.17)	**0.014**

Bold indicates *P*-values less than 0.05

*Continuous variables are expressed as mean ± standard deviation. Mann-Whitney U test was used to test differences between two groups. Categorical data were described using frequency and percentage, using Pearson χ2 test, Fisher exact 2-sided test

### Secondary outcomes

At baseline, there was no difference in the MOCA scores in the sham and PEMS groups (24.90 ± 2.82 vs 24.89 ± 3.16, *P* = 0.936, respectively) (Table 1), and the percentage of patients with MoCA scores < 26 was not different between the PEMS and sham groups [57.60% (72/125) vs 54.89% (73/133), *P* = 0.661, respectively]. At the end of the six-month intervention period, the MoCA scores improved in both sham and PMES groups, the percentage of patients with MoCA scores < 26 was lower in PEMS group than in the sham group [12.00%(15/125) vs 24.06%(32/133), *P* = 0.012, respectively], MoCA scores in the PMES group were higher than those in the sham group at 6 months (28.26 ± 1.95 vs 27.26 ± 2.20, *P* < 0.001, respectively), and the mean value of the MoCA score change (M6-baseline) was higher in the PMES group (3.50 ± 2.55) than in the sham group (2.72 ± 2.52,*P* = 0.005) (Table 4).

**Table 4:** The mean change in MoCA Score and HRSD in Sham and PMES groups

	Sham group (133)	PMES group (125)	P*
MoCA Score, (Mean SD)	2.72 ± 2.52	3.50 ± 2.55	**0.005**
HRSD Score, (Mean SD)	−10.48 ± 6.10	−11.93 ± 5.32	**0.036**

Bold indicates *P*-values less than 0.05

*Continuous variables are expressed as mean ± standard deviation. Mann-Whitney U test was used to test differences between two groups

### Adverse reactions and compliance

There were no adverse reactions reported in either the PMES group or the sham group during the treatment period. The mean number of applications of the devices over the 6 months was 166 (92.22%) in the PMES group and 159 (88.33%) in the sham group. The difference between the two groups was not significant (*P* = 0.213).

## Discussion

The primary and secondary outcomes of this randomized, sham-controlled study showed that daily treatment with PMES in combination with pharmacotherapy was more effective than pharmacotherapy with sham stimulation in PSD. The results of the study showed that PMES and sham treatment were both effective in improving PSD and cognition. At the end of the 6-month follow-up period, the decreases in HRSD scores and the percentages of patients showing a treatment response and depression remission were smaller in the sham group than in the PMES group. In addition to the improvements in PSD, the secondary outcome, the MoCA score, also showed a significant increase in the two groups. The increased in MoCA scores were lower and the percentage of patients with a MoCA score <26 was higher in the sham group than in the PMES group at 6 months.

The incidence of PSD is very high. PSD affects 12–72% of stroke patients [31, 32]. A meta-analysis showed that 31% of patients developed depression within 5 years after stroke. In the past, physical disability caused by stroke was often the focus of treatment. However, in recent years, the treatment of psychological comorbidities, which influence patient rehabilitation, has also attracted the attention of clinicians. After stroke, many patients suffer motor impairment, which limits their mobility, and lose confidence, which may lead to PSD [33]. Previous studies have proven the positive effects of PEMS on motor function [25, 26]. Animal experiments have shown that FNS alone or in combination with drug therapy could improve PSD [34], while in clinical practice, the effects of PMES on PSD have been unclear. Hence, in the present study, we investigated the effects of PMES on PSD assessed by the HRDS, and we found that PMES combined with antidepressants was significantly more successful in improving poststroke depression than medication alone. In this study, during the 6-month follow-up period, a higher percentage of patients with HRSD scores of ≤9 and ≥ 50% reduction in the PMES group than in the sham group showed that more patients from the PMES group had lower levels of depression. The effects of sham stimulation in this study might have involved the use of antidepressants during the treatment period. From every outcome measure, treatment effects of the PMES was much better than that of the sham stimulation. Therefore, the improved effects with PMES treatment in PSD was mainly derived from the PMES treatment itself.

Cognitive impairment is a common sequelae after stroke. The cerebellum plays a role in cognition [35, 36]. Stroke can affect cerebellar function and produce vascular dementia (VD). A previous study found that activation of the cerebellum significantly alleviated VD, and poststroke cognitive impairment was improved by FNS treatment [17]. Fan et al. found that cognitive function decreased 2 months after chronic cerebral hypoperfusion and was worse 4 months after hypoperfusion, and the cognitive function improved after FNS treatment [17]. Although animal studies have shown that PMES could improve cognitive function after cerebral ischaemia, there is limited information about the role of PMES in cognitive impairment after stroke in clinical studies. In our study, we observed that PMES could improve cognition in ischaemic stroke patients, the mean value of the MoCA score change was higher in the PMES group than in the sham group, and the percentage of patients with MoCA scores < 26 in PEMS group was lower than in sham group.

The exact mechanism of action of PMES is unclear. According to previous studies, FNS could upregulate NE and 5-HT in the frontal lobes of rats with depression [37, 38], in addition, the positive affective state or enhanced arousal and attention could improve cognition, which seems to be a plausible mechanism [39].

Some limitations of this study merit consideration. First, NIHSS scores have been shown to correlate with infarction volume, and we lacked data on infarction volume. Second, the peak current was 10 mA for PMES and 0.18 mA for sham stimulation, which might have given patients clues about group assignment and had an effect on the experimental results. Third, each group was prescribed and reported taking antidepressants during the 6-month treatment period, and the doses and type of drug were not standardized. In addition, cognitive status was assessed using the MoCA, but the scores on this questionnaire are also affected by education level, and the cutoff value was not adjusted for people with low literacy, which may have influenced the results. This is a limitation of the study but represents the context of everyday practice.

## Conclusions

In conclusion, our findings indicated that PMES adjunctive to antidepressant therapy is effective in reducing depression and achieving remission in the short term. We also demonstrated that improved poststroke depression was associated with improved cognition. These data indicate that PMES may be a safe and low-cost therapy to improve clinical stroke outcomes.

## Data Availability

Data used in this study may be available by request to corresponding author via email: 531324679@qq.com

## Abbreviations

PMES: Percutaneous mastoid electrical stimulator
HRSD: Hamilton Rating Scale for Depression
MoCA: Montreal Cognitive Assessment
PSD: Poststroke depression
FNS: Fastigial nucleus stimulation
CVFT: Cerebrovascular function therapy
NIHSS: National Institutes of Health Stroke Scale
